# Supplementary material for: Complexome profiling of the Chlamydomonas psb28 mutant reveals TEF5 as an early PSII assembly factor
Source: Plant Cell. 2025 Mar 17;37(6):koaf055. doi: 10.1093/plcell/koaf055 (PMC12164590; doi:10.1093/plcell/koaf055)
Supplement: koaf055_Supplementary_Data [file koaf055_supplementary_data.zip › psb28_interactive.zip!/plots/intensity/qualityPlots/peptides/CPL6_Cre06.g279500.t1.1.html]

### Cre06.g279500.t1.1

Trivial name: CPL6  
SeqCoverage [median]: 25.50  
Mass: 32.51 kDa  
  
 

| Sequence | MissedCleavage | Mass [Da] | wtIntensity | muIntensity | Score |
| --- | --- | --- | --- | --- | --- |
| EQEQEQEKYVSALPLMPPLSQK | 1 | 2571.29 | 0.00 | 3421.51 | 55.01 |
| IFLLMEEVRR | 1 | 1304.73 | 11013.28 | 31710.88 | 65.35 |
| LGFGGTTYLEFVQSMHLPR | 0 | 2152.08 | 1124.96 | 0.00 | 55.44 |
| QLATEHDPR | 0 | 1065.52 | 0.00 | 4815.18 | 74.46 |
| YVSALPLMPPLSQK | 0 | 1542.85 | 1768.52 | 16404.46 | 104.52 |
|  |  |  | 13906.8 | 56352.0 |  |
